# Correlation of post-neoadjuvant chemotherapy response on MRI with final histology in breast cancer patients

**DOI:** 10.1186/bcr3768

**Published:** 2015-11-05

**Authors:** Anjum Mahatma, Lyn Jones, Alexandra Valencia

**Affiliations:** 1 Breast care centre, North Bristol NHS Trust, Bristol, UK

## Introduction

We used MRI breasts to assess neoadjuvant chemotherapy response in line with departmental protocol. The aims were to see the correlation of findings on MRI with final histology in patients with breast cancer receiving neoadjuvant chemotherapy, and to assess the accuracy of our reporting and to evaluate the cause for any discordancy.

## Methods

Retrospective data collection from March 2012 to May 2014. Data on 50 consecutive patients, who had both pre and post neoadjuvant MRIs, were collected. Use of CRIS, NBT PACS and UHB PACS for reports and image visualisation. Use of Ultra inquires on the intranet for histopathology reports.

## Results

Discrepancy in size of residual tumour between MRI and histology within 10 mm was considered concordant. Concordant size between MR and histology = 31/50 (62%). Discordant size between MR and histology = 19/50 (38%). For complete response: sensitivity = 46%, specificity = 86%, positive predictive value = 71% (95% CI 45−88%), negative predictive value = 70% (95% CI 53−82%), accuracy = 70%.

## Conclusion

Good specificity but low sensitivity in line with published literature. The time interval between second MRI and time to surgery did not affect the ability of MRI to predict response. Presence of DCIS and LCIS (42%) influenced MRI-histology discrepancy. Hormonal-positive, Her2-positive and triple-positive were more likely to show size discrepancy compared with other hormone profiles.

## Recommendation

We plan to integrate DWI into our reports to increase sensitivity.

